# Long non-coding RNA NEAT1 confers oncogenic role in triple-negative breast cancer through modulating chemoresistance and cancer stemness

**DOI:** 10.1038/s41419-019-1513-5

**Published:** 2019-03-20

**Authors:** Vivian Yvonne Shin, Jiawei Chen, Isabella Wai -Yin Cheuk, Man-Ting Siu, Chi-Wang Ho, Xian Wang, Hongchuan Jin, Ava Kwong

**Affiliations:** 1 Department of Surgery, The University of Hong Kong, Pokfulam, Hong Kong; 2 Department of Medical Oncology, Sir Run Run Shaw Hospital, Medical School of Zhejiang University, Hangzhou, China; 3 Department of Medical Oncology, Key Laboratory of Biotherapy in Zhejiang, Sir Runrun Shaw Hospital, Medical School of Zhejiang University, Zhejiang, China; 4 Department of Surgery, The Hong Kong Sanatorium and Hospital, Happy Valley, Hong Kong; 5 The Hong Kong Hereditary Breast Cancer Family Registry, Shau Kei Wan, Hong Kong

## Abstract

Triple-negative breast cancer (TNBC) is a malignant subtype of breast cancer with the absence of targeted therapy, resulting in poor prognosis in patients. Chemotherapy remains the mainstay of treatment for TNBC; however, development of drug resistance is the main obstacle for successful treatments. In recent years, long non-coding RNA (lncRNA) has been implicated in multiple biological functions in various diseases, particularly cancers. Accumulating evidence suggested that lncRNA nuclear paraspeckle assembly transcript 1 (NEAT1) expression is dysregulated in many human cancers and thus is a useful prognostic marker for cancer patients. Nevertheless, the mechanism of how NEAT1 confers drug resistance in TNBC is still largely unknown. We performed lncRNA profiling by the LncRNA Profiler qPCR Array Kit in normal control (NC) and breast cancers (BC) blood samples and further validated in a larger cohort of samples by qRT-PCR. Gene expression level and localization were investigated by qRT-PCR, western blotting, and immunofluorescence staining. Flow cytometric analysis was carried out to detect cancer stem cells. Functional studies were performed both in vitro and in vivo xenograft model. Among 90 lncRNAs, NEAT1 was highly expressed in the blood samples of breast cancer patients than in NC. In particular, the expression of NEAT1 was higher in TNBC tissues than other subgroups. Functional studies revealed that NEAT1 conferred oncogenic role by regulating apoptosis and cell cycle progression in TNBC cells. We identified that knockdown of NEAT1 sensitized cells to chemotherapy, indicating the involvement in chemoresistance. Importantly, shNEAT1 reduced stem cell populations such as CD44+/CD24−, ALDH+, and SOX2+, implicating that NEAT1 was closely related to cancer stemness in TNBC. Our data highlighted the roles of NEAT1 chemoresistance and cancer stemness, suggesting that it could be used as a new clinical therapeutic target for treating TNBC patients especially those with drug resistance.

## Introduction

Tremendous advances in human genomics during past decades have unravelled the transcriptional landscape that is far more complex than what we originally expected. There are >80% of the human genome transcribed^1,2^, however, <2% of the transcribed genome codes for protein and the remaining genome consists of non-coding RNAs (ncRNAs). Extensive efforts have been made in this field and discovered >3000 ncRNAs with known functions to date^3^. Long non-coding RNAs (lncRNAs) is a subgroup of ncRNAs characterized as transcripts >200 nucleotides that are not translated into protein which distinguish from other short ncRNAs such as microRNAs (miRNAs), short interfering RNAs (siRNAs), etc^4^. LncRNAs play critical roles in gene expression regulation on both transcriptional and posttranscriptional levels, resulting in a wide spectrum of biological processes including tumor initiation, growth and metastasis in different human diseases including cancers^5–7^. The nuclear-enriched abundant transcript 1 (NEAT1), which is a recently discovered essential component of nuclear paraspeckles^8,9^, is identified to be dysregulated in various solid cancers^10,11^. Under most circumstances, NEAT1 functions as a oncogene in different malignancies, such as lung cancer, oesophageal squamous cell carcinoma, laryngeal squamous cell carcinoma, ovarian cancer, colorectal cancer, hepatocellular carcinoma, prostate cancer, and glioma^12–18^. On the contrary, there are studies that reported the tumor-suppressive role of NEAT1 in other cancers, such as acute promyelocytic leukemia^19^. Also, it was reported to be regulated by p53 for tumor transformation suppression in pancreatic cancer^20^ and low expression of NEAT1 was correlated with poor prognosis in colon, lung, and breast cancers^21^.

Breast cancer is one of the most leading causes for cancer-related death among women worldwide, and the incidence rate is still increasing. Triple-negative breast cancer (TNBC), which lack the expression of estrogen receptor (ER), progesterone receptor (PR), and human epidermal growth factor receptor 2 (HER2), is an aggressive subtype of breast cancer with higher risk of early relapse and poor prognosis when compared with other subtypes^22^. By now, only limited studies reported on the action of NEAT1 in breast cancer, and others focused on its implication as a hypoxia-induced lncRNAs and led to accelerated cellular proliferation and increased tumorigenesis^23^. For example, NEAT1 promoted breast cancer growth by regulating miRNAs, such as miR-548^24^ and miR-448^25^. The FOXN3-NEAT1-SIN3A complex promoted epithelial-to-mesenchymal transition and invasion of breast cancer cells^26^. In recent years, there has been convincing evidence of lncRNAs in the regulation of stem cell properties^27–29^; however, the mechanism involving cancer stem cells in relation to chemoresistance is still unclear. This study aims to examine the functional role of NEAT1 in stemness features and chemoresistance in TNBC.

## Results

### High expression of NEAT1 in breast cancer patients

**Fig. 1:**
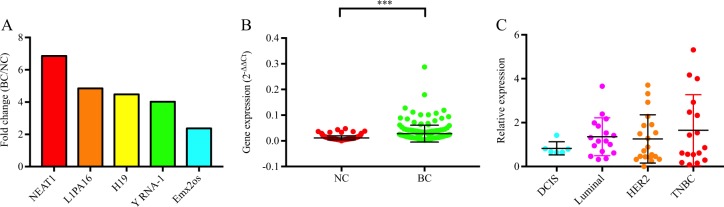
Nuclear paraspeckle assembly transcript 1 (NEAT1) expression in breast cancer patients. **a** The top 5 upregulated long non-coding RNAs in breast cancer patients; **b** NEAT1 was upregulated in peripheral blood of breast cancer (BC) patients when compared with normal controls (NC); **c** NEAT1 expression in tissues was higher in triple-negative BC than in other subtypes. ***P < 0.001 indicates statistically different from the control

Based on our microarray finding, we identified several dysregulated lncRNAs that are highly expressed in breast cancer patients. Among all, NEAT1 is the top upregulated lncRNA with a 6.86-fold increase (Fig. 1a). Further validation was performed in the circulation of 192 normal controls and 179 breast cancer patients by quantitative reverse transcription –PCR (qRT-PCR). The expression level of NEAT1 were significantly higher in breast cancer than in normal controls (Fig. 1b). We further evaluated the expression of NEAT1 in paired breast cancer tissues and stratified into ductal carcinoma in situ (DCIS), luminal, HER2, and TNBC subtypes. Results showed that NEAT1 expression was more prominent in TNBC than other subtypes (Fig. 1c), implicating an important role of NEAT1 in the carcinogenesis of TNBC. Since it is well known that NEAT1 has two isoforms (NEAT1-1 and NEAT1-2), we further investigated which specific isoform is the major form in TNBC. qRT-PCR result showed that there was no significant difference in the expression of NEAT1-2 among subtypes including DCIS, luminal, HER2 and TNBC, suggesting that NEAT1-1 is the major form in TNBC (Supplementary Fig 1).

### NEAT1 is required for G1-phase cell cycle arrest and apoptosis

**Fig. 2:**
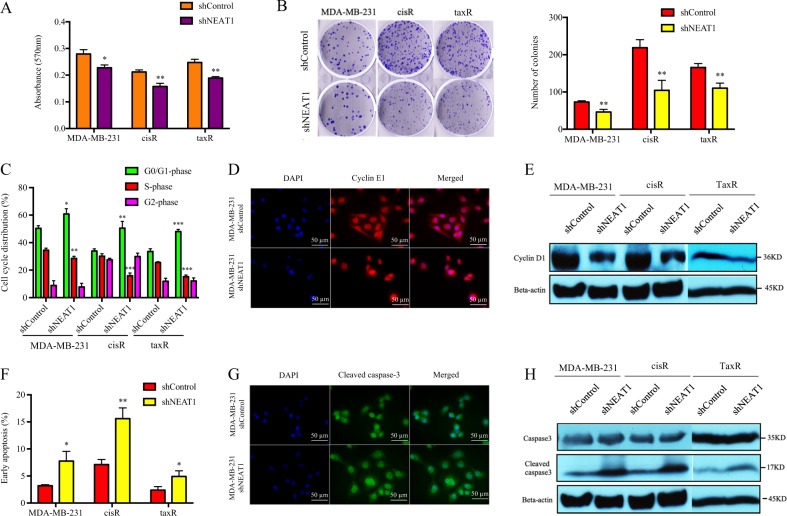
Nuclear paraspeckle assembly transcript 1 (NEAT1) is required for breast cancer cell growth. **a** NEAT1 knockdown in triple-negative breast cancer cells led to significant decreased cell viability; **b** colony-formation ability was significantly impeded in NEAT1 stable knockdown cells; **c** NEAT1 silencing led to G1-phase cell cycle arrest and increased S-phase cell population; **d** Immunofluorescence staining and **e** western blotting showed that cyclin E1 and cyclin D1 were decreased upon NEAT1 knockdown; **f** shNEAT1 significantly enhanced early apoptosis and cleaved caspase-3 was increased in **g** immunofluorescence staining and **h** western blot analysis. *P < 0.05; **P < 0.01 indicates statistically different from the control

We developed NEAT1 stable knockdown in MDA-MB-231 and its cisplatin/taxol resistance clone, MDA-MB-231/cisR/taxR, to study the tumor-promoting effect of NEAT1 in TNBC. Significant inhibition of cell proliferation and colony formation was observed in shNEAT1 in both MDA-MB-231 and cisR/taxR (Fig. 2a, b). To understand the functional mechanism of growth inhibition, Annexin V binding assay was performed to detect apoptosis in shNEAT1 cells. Cell cycle analysis revealed a G1-phase arrest and increased S-phase cell population in all shNEAT1 cells (Fig. 2c). Knockdown of NEAT1 reduced the expression of cyclin E1 and D1 (Fig. 2d, e), indicating that NEAT1 is required in cell cycle progression. Besides, early apoptosis was significantly increased in shNEAT1 cells (Fig. 2f), which was concomitant with increased cleaved caspase-3 by immunofluorescence staining and western blot analysis (Fig. 2g, h). All these data showed that blocking of NEAT1 had a more prominent effect on cell proliferation and apoptosis in cisR and taxR cells when compared with parental cells.

### NEAT1 mediated drug resistance and cancer stemness

**Fig. 3:**
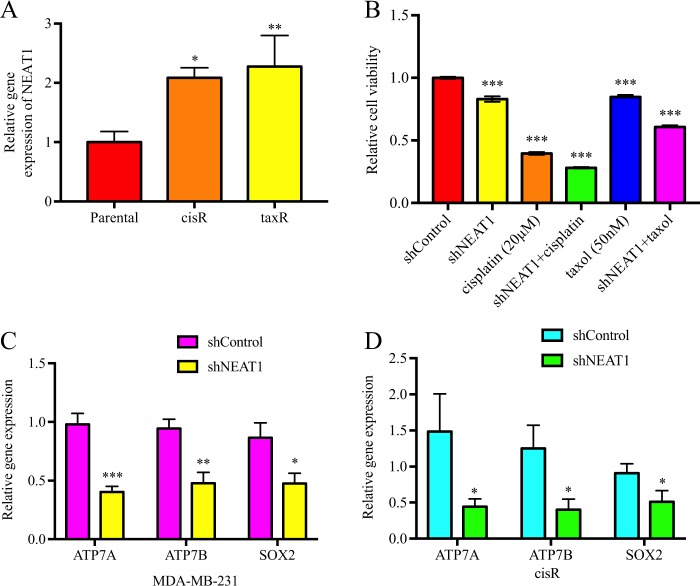
Nuclear paraspeckle assembly transcript 1 (NEAT1) played important role in drug resistance. **a** NEAT1 expression was upregulated in cisplatin (cisR) and taxol (taxR) resistant cells; **b** NEAT1 knockdown had synergistic effect with chemotherapeutic drugs by inhibiting cancer cells growth; **c**, **d** ATP7A/7B and SOX2 expression were decreased in shNEAT1 cells. *P < 0.05; **P < 0.01; ***P < 0.001 indicates statistically different from the control

**Fig. 4:**
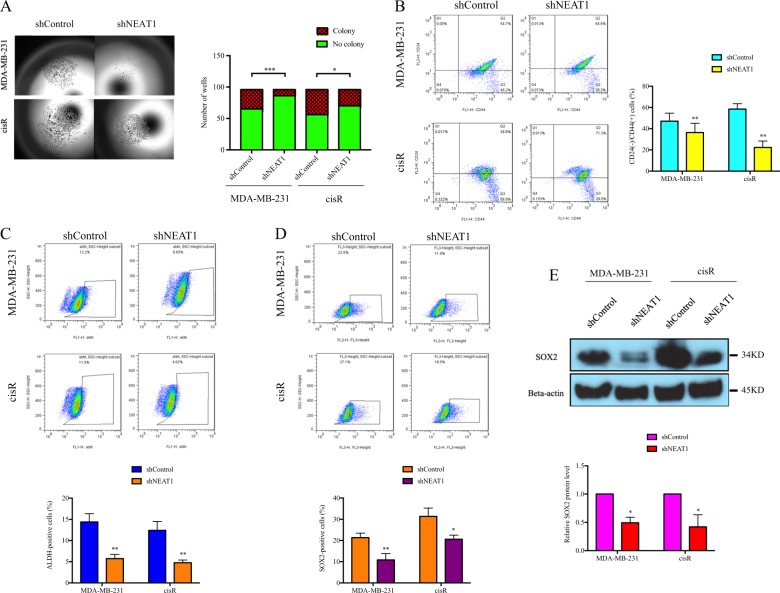
Nuclear paraspeckle assembly transcript 1 (NEAT1) was related to stemness in triple-negative breast cancer. **a** NEAT1 knockdown significantly impeded single-cell clonogenic ability; NEAT1 silencing decreased **b** CD24−/CD44+; **c** ALDH+, and **d** SOX2+ cells populations by flow analysis; **e** western blot demonstrated that SOX2 protein level was decreased in shNEAT1 cells. *P < 0.05; **P < 0.01; ***P < 0.001 indicates statistically different from the control

As NEAT1 expression is upregulated in cisR and taxR compared with its parental cells (Fig. 3a), suggesting its role in drug resistance, we further investigated the effect of NEAT1 on drug sensitivity. NEAT1 knockdown further decreased cell growth in cisplatin- or taxol-treated cells, demonstrating a synergistic effect of the combination of shNEAT1 and chemotherapeutic drugs in breast cancer (Fig. 3b). qRT-PCR results identified that drug transporter genes, such as ATP7A and ATP7B, were downregulated in shNEAT1 cells (Fig. 3c, d). Interestingly, a stemness marker, SOX2, was also downregulated in shNEAT1 cells (Fig. 3c, d). In single-cell clonogenic assays, significant fewer number of colonies and smaller colony size were observed in shNEAT1 cells (Fig. 4a), suggesting an impeded self-renewal ability. Next, flow cytometric analysis was used to quantify the cancer stem cell marker-positive cell population. CD44+/CD24−, ALDH+, and SOX2+ cell population decreased significantly in shNEAT1 cells (Fig. 4b–d) and SOX2 protein level was also downregulated by the loss of NEAT1 (Fig. 4e). These data illustrated that NEAT1 regulated cancer cell markers to regulate cell proliferation.

### NEAT1 silencing inhibited tumor growth in vivo

**Fig. 5:**
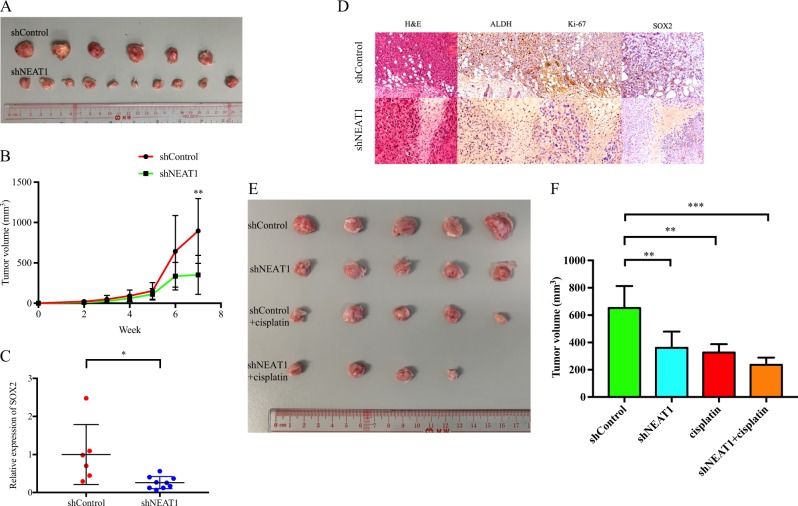
Nuclear paraspeckle assembly transcript 1 (NEAT1) knockdown inhibited tumor growth in vivo. **a** Gross appearance of xenograft tumors in different groups of mice; **b** the tumor volume was smaller in shNEAT1-treated mice; **c** SOX2 mRNA expression was significantly downregulated in shNEAT1-treated xenograft tumors; **d** representative images of tumor sections on hematoxylin and eosin, aldehyde dehydrogenase, Ki-67, and SOX2 staining; **e** gross appearance of xenograft tumors; and **f** tumor volume in four different groups of mice; *P < 0.05, **P < 0.01, ***P < 0.001 indicates statistically different from the control

To determine the effect of NEAT1 on breast cancer tumorigenicity in vivo, NOD-SCID mouse xenograft tumor model was used. shNEAT1 cells were injected subcutaneously into mammary fat pad of mice. Macroscopic observation of the injected mice revealed that tumor volume was smaller in shNEAT1-treated mice than in the shControl-treated mice (Fig. 5a, b). Meanwhile, aldehyde dehydrogenase (ALDH) and SOX2 expression levels were significantly lower in the shNEAT1-treated tumors compared with the shControl tumors (Fig. 5c, d). To further elucidate the role of NEAT1 in chemoresistance in in vivo model, cisplatin was administered locally in shControl- and shNEAT1-treated mice. Result showed that the tumor volume in the shNEAT1+cisplatin group was smaller than in the shNEAT1-treated group, indicating that NEAT1 suppression enhanced cisplatin sensitivity in breast cancer (Fig. 5e, f). Taken together, our results suggested that NEAT1 contributed in tumorigenesis of TNBC through regulating cancer stemness and chemoresistance.

## Discussion

LncRNAs has long been considered as “transcriptional noise” as they do not code for proteins^30^. Currently, lncRNAs are now accepted as important regulators in cancer development and different biological processes such as chromatin remodeling and transcriptional and posttranscriptional regulation^30^. Enormous evidence showed that NEAT1 upregulation contributed to the progression of many malignancies^31^. A pan-cancer lncRNA analysis has shown that NEAT1 is overexpressed in tumor tissues, including bladder urothelial carcinoma, cervical squamous cell carcinoma, cholangiocarcinoma, colon carcinoma, esophageal carcinoma, head and neck squamous cell carcinoma, kidney clear cell carcinoma, hepatocellular carcinoma, pancreatic cancer, prostate cancer, gastric cancer, and thyroid cancer^32^. On the contrary, only few reports suggested the tumor-suppressive role of NEAT1, such as leukemia^19^. In breast cancer, high expression of NEAT1 was correlated with poor overall survival of ER+ breast cancer patients. Besides, interaction of FOXN3 with NEAT1/SIN3A showed to repress GATA3 in breast cancer metastasis^26^. In addition, functions of NEAT1 has been implicated in cell growth, migration, and invasion and regulated miR-488 and ZEB1^25^. Patients with high NEAT1 expression in tumors correlated with poor survival^23^. Hypoxia-inducible factor-2 caused aggregation of nuclear paraspeckles in hypoxia condition, which induced pro-tumorigenic in breast cancer. Although recent studies revealed the crucial roles for lncRNAs in the breast cancer carcinogenesis^24,26,33^, the exact molecular mechanism underlying breast cancer is still unclear especially in TNBC. Strikingly, some studies revealed that NEAT1 could be detected in the circulation and was dysregulated in peripheral blood mononuclear cells in several cancers; these suggested that NEAT1 is a potential diagnostic and prognostic biomarker in cancers^34,35^. In our study, we identified that NEAT1 expression was upregulated in the circulation of breast cancer patients and remarkably discriminated from normal controls. We are the first to report that NEAT1 is upregulated in TNBC and expand our understanding on the role of NEAT1 in this malignant subtype of breast cancer. The inhibitory effect of NEAT1 knockdown caused reduction in cell proliferation, colony-forming ability, induction of apoptosis, and cell cycle arrest. This was also seen in ovarian cancer that NEAT1 suppressed apoptosis and increased S-phase cell population through regulating Bcl2 that is mediated by miR-34a-5p^36^. In pancreatic cancer, NEAT1 facilitated its oncogenicity by directly binding to miR-506-3p, the miRNA that has been reported as a tumor suppressor in diverse cancers^37^. Similar findings that NEAT1 functioned as tumor suppressor were reported in other cancers such as non-small cell lung cancer^38^, hepatocellular carcinoma^39^, and glioma^40^. All these findings put forward the dual action of NEAT1 in carcinogenesis.

In concordance with our findings, there was evidence highlighting the involvement of NEAT1 in chemoresistance. In ovarian cancer, NEAT1 contributed to paclitaxel resistance partly through upregulating ZEB1 expression by sponging miR-194^41^. In cisplatin-resistant gastric cancer cells, NEAT1 expression was significantly upregulated accompanied with the induction of P-glycoprotein, which is a multidrug resistance-associated protein^42^. NEAT1 is also reported to contribute to docetaxel resistance through inducing RET expression by sponging miR-34a in prostate cancer^43^. In nasopharyngeal carcinoma, NEAT1 enhanced the cisplatin resistance by targeting Rsf-1 and Ras-mitogen-associated protein kinase signaling pathway^44^. We revealed that the combination of chemodrug (cisplatin or taxol) and NEAT1 knockdown synergistically inhibited drug sensitivity when compared with cisplatin or taxol alone. Overexpression of NEAT1 in cisplatin- and taxol-resistant cells indicated its role chemoresistance in TNBC. Downregulation of drug transporter genes in shNEAT1 cells suggested a potential NEAT1-mediated chemoresistance mechanism. During the past decade, very few studies delineated on the role of lncRNAs in stem cell biology^45–47^. Silencing of NEAT1 reduced cancer stem cell-like properties in lung cancer stem cells and CTR1 expression was negatively correlated with NEAT1 expression^48^. Furthermore, NEAT1 was upregulated in glioma stem cells, and inhibition of NEAT1 retarded cell proliferation, migration, and invasion^49^. Lo et al. reported that in DCIS, NEAT1 was functionally required for maintaining stemness such as anchorage-independent growth in soft agar, self-renewal ability, and sphere-formation assay^50^. In conclusion, we found that NEAT1 was overexpressed in the circulation of breast cancer, especially TNBC. The tumor-promoting effect was due to dysregulated apoptosis and cell cycle. We identified for the first time that NEAT1 is associated with chemoresistance and cancer stem cell property in TNBC. These findings provide evidence that NEAT1 is a potential therapeutic target to overcome chemoresistance for treating TNBC.

## Materials and Methods

### Clinical specimen

Breast cancer patients were recruited through Queen Mary Hospital, Tung Wah Hospital, and Hong Kong Sanatorium and Hospital through the Hong Kong Hereditary Breast Cancer Family Registry. This study was approved by Institutional Review Board of the University of Hong Kong (UW 15-441). All participants of this study including breast cancer patients and normal controls without personal history of cancers have agreed and signed the consent form. Blood samples from 179 breast cancer patients and 192 normal controls were included and the patients’ demographic characteristics such as age, histological type, staging, metastasis, and subtypes are listed in Table 1.

**Table 1:** Clinical characteristics of breast cancer patients

	Breast cancer (*n* = 179)
Age [years; mean (SD)]	56.7 (11.7)
Histological type	
DCIS	16
IDC	146
ILC	5
Others	12
Bilateral cancer	5
Distant metastasis	
Before Surgery	1
After Surgery	11
Stage	
0	26
I	75
II	55
III	23
IV	1
Subtypes	
Luminal	154
HER2	17
TNBC	11
NA	4

*DCIS* ductal carcinoma in situ, *IDC* invasive ductal carcinoma, *ILC* invasive lobular carcinoma, *HER2* human epidermal growth factor receptor 2, *TNBC* triple-negative breast cancer, *NA* not available

### LncRNA expression profiling

LncProfilers™ qPCR Array Kit (System Biosciences, CA, USA) containing 90 lncRNAs was used to compare the lncRNA expression levels in breast cancer patients and normal controls. RNA from peripheral blood from five normal controls and five breast cancer patients were isolated using the QIAamp RNA Blood Mini Kit (Qiagen, CA, USA). After total RNA was extracted, lncRNAs were tagged with polyA tail and annealed to adaptor using the LncProfilers™ qPCR Array Kits. After the conversion of cDNA, qPCR assays were conducted. The relative amount of each lncRNA in normal controls compared to that in breast cancer was described using the 2^−ΔΔCT^ method.

### RNA extraction and qRT-PCR

Total RNA was extracted by using the QIAamp RNA Blood Mini Kit according to the manufacturer’s standard instruction. Briefly, QIAzol Lysis Reagent was added to peripheral blood sample. After vortex homogenization, chloroform was used for separation followed by absolute ethanol to precipitate RNA. The eluted RNA concentration was quantified by NanoDrop 1000 (Thermo Scientific, DE, USA). Reverse transcription was performed by the High Capacity cDNA Reverse Transcription Kit (Thermo Fisher Scientific, CA, USA). LightCycler® 480 SYBR Green I Master (Roche, OR, USA) was used in the Roche LightCycler® 480 System to perform qRT-PCR.

### Cell culture and transfection

The metastatic TNBC cell line MDA-MB-231 (ATCC® HTB-26™) was purchased from the American Type Culture Collection and cultured in RPMI 1640 medium (Invitrogen, Grand Island, USA) supplemented with 10% heat-inactivated fetal bovine serum. cisR or taxR sub-line of MDA-MB-231 cells were established by chronic treatment with cisplatin- or taxol-supplemented (Sigma-Aldrich, MO, USA) medium. The IC_50_ of cisplatin were 23.56 and 126.5 μM in MDA-MB-231 and cisR cells (Supplementary Fig 2A), while the IC_50_ of taxol were 57.63 and 544.2 nM in MDA-MB-231 and taxR cells (Supplementary Fig 2B), confirming the resistance characteristics of these two in-house developed cell lines. To develop stable negative control or NEAT1 knockdown clones, cells were transfected with negative control shRNA or NEAT1 shRNA expression vector with Lipofectamine 3000 Reagent (Thermo Fisher Scientific, CA, USA). The shRNA duplexes designed against NEAT1 (Gene ID: 283131) with the target sequence (CATGGACCGTGGTTTGTTACT) synthesized by GenePharma Company (Shanghai, China) were incorporated into the pGPU6/GFP/Neo-shRNA vector to get pGPU6/GFP/Neo-shRNA-NEAT1. Cells were transfected with 1 µg shRNA expression plasmids and were selected with 1 mg/ml Geneticin (Thermo Fisher Scientific, CA, USA) starting from 3 days after transfection.

### Cell proliferation assay

Thiazolyl blue tetrazolium bromide (MTT)-based assay was used to quantify cell viability after 3 days of cisplatin or taxol treatment. Briefly, 5 × 10^3^ cells were seeded onto a 96-well plate and the medium was replaced with complete medium supplemented with 3% MTT and incubated for 2 h. Then the intracellular purple formazan crystal products were dissolved in 100 μl of dimethyl sulfoxide (Sigma-Aldrich, MO, USA) followed by the colorimetric product measured by Multiskan™ FC Microplate Photometer (Thermo Fisher Scientific, CA, USA) at 570 nm.

### Apoptosis assay

Fluorescein isothiocyanate (FITC) Annexin V Apoptosis Detection Kit (BD Biosciences, USA) was used to detect the apoptotic cell population according to manufacturer’s standard protocol. Briefly, cells were collected and suspended in 1× Annexin V binding buffer at 1 × 10^6^ cells/ml density. In all, 100 μl of resuspended cells were stained with 5 μl of FITC Annexin V and 5 μl of propidium iodide (PI). The mixture was vortexed at room temperature in dark for 15 min. Then the cells were washed and resuspended in 400 μl of 1× Annexin V-binding buffer and analyzed by BD FACSCalibur. At least 10,000 gated cells were counted in each sample for analysis calculation.

### Cell cycle analysis

To determine the effect of NEAT1 knockdown on cell cycle distribution, cellular DNA contents were stained with PI for flow cytometric analysis. Briefly, cells were collected, washed with chilled phosphate-buffered saline (PBS), and fixed with ice-cold 70% ethanol overnight. The fixed cells were treated with 20 μg/ml of PI and 0.2 mg/ml of RNase A (Thermo Fisher Scientific, CA, USA) for 40 min in dark. After PI staining, cells were resuspended in 500 μl of PBS and subjected for flow cytometric analysis by using the BD FACSCalibur platform.

### ALDH assay

ALDH activity was quantified using the Aldefluor Assay Kit (StemCell Technologies Vancouver, Canada) according to the manufacturer’s instructions. Briefly, cells were resuspended in Aldefluor assay buffer containing the ALDH substrate bodipy-aminoacetaldehyde for 45 min at 37 °C. In parallel, equal amount of cells were incubated with diethylaminobenzaldehyde (DEAB), which is an ALDH inhibitor to serve as negative control. Cells were washed with PBS and resuspended in Aldefluor assay buffer before subjected to flow analysis by BD FACSCalibur. ALDH-positive cell population was determined relative to the corresponding DEAB-treated negative control group. The acquired data were analyzed with the FlowJo software 7.6.1 (TreeStar, USA).

### Colony-formation assay

MDA-MB-231 and cisR cells with stable NEAT1 knockdown or empty vector were seeded on 6-well plate at a density of 500 cells/well. After incubation for 7 days, 1 ml of methanol with 1% crystal violet (Sigma-Aldrich, MO, USA) was added to each well and stained for 30 min. Photographs were taken from each well, and the colonies were counted with the colony-forming unit (CFU) software OpenCFU v3.9.0 (Geissmann) under the same condition.

### Clonogenic assay

The in vitro self-renewal ability of stem cell was reflected by the anchorage-dependent colony formation. Briefly, single cell was seeded in 96-well plate and incubated for 7 days. Single-cell clonogenic ability was evaluated by the number of cell colonies (no less than 50 cells) formed.

### Western blotting

Cells were trypsinized and washed with cold PBS twice and lysed in 100 µl of lysis buffer (Cell Signaling Technology, MA, USA) for 20 min on ice. Samples were then centrifuged for 15 min at 14,000 × *g* at 4 °C. Protein concentration was determined by the Bradford assay (Bio-Rad, CA, USA). In all, 60 µg of protein was loaded into 10–15% sodium dodecyl sulfate (SDS) gel and fractionated by SDS-polyacrylamide gel electrophoresis and transferred to a polyvinylidene difluoride membrane using a transfer system. The membranes were blocked with 5% non-fat milk in tris buffered saline with Tween-20 (TBST) to block the unspecific binding sites for 60 min at room temperature. The membranes were then washed with TBST and incubated with antibodies against cyclin D1 (1:1000), cleaved caspase-3 (1:1000), caspase-3 (1:1000), β-actin (1:2000), SOX2 (1:1000), and CD44 (1:1000) at 4 °C overnight with gentle shaking. The membranes were washed with TBST three times and incubated with horseradish peroxidase-conjugated anti-mouse or anti-rabbit antibodies (1:2000) for 60 min at room temperature. Blots were developed with enhanced chemiluminescence system (Amersham Biosciences, Buckinghamshire, UK) and quantified using ImageJ 1.52a (Wayne Rasband, National Institute of Health).

### In vivo xenograft animal model

The animal experiment ethics (CULATR 4409-17) was approved by the Committee on the Use of Live Animals in Teaching and Research in the University of Hong Kong. Six-week-old female NOD-SCID mice were used in animal models. Briefly, 2 × 10^6^ MDA-MB-231 cells with shNEAT1 or shControl suspended in 100 µl of PBS were injected into mammary fat pad of the mice. Tumor volumes were measured from 2 weeks onwards with palpable mass by using formula [1/2(length × width^2^)] every 7 days. In the cisplatin treatment group, cisplatin was administered locally at the tumor mass for 2 weeks after the tumor volume reached 100 mm^3^ at a dose of 3 mg/kg twice a week.

### Immunohistochemistry staining

Briefly, antigen unmasking was performed after deparaffinization. The sections were incubated with primary antibodies (Cell Signaling, MA, USA; Abcam, MA, USA) in recommended diluent overnight at 4 °C. The detection was performed according to the manufacturer’s instructions using Signal Stain Boost Detection Reagent (Cell Signaling) and incubated in a humidified chamber for 30 min at room temperature followed by washing. Then Signal Stain DAB was added to each section and subjected to dehydration of the sections by using 95% and 100% ethanol, respectively, before mounting.

### Statistical analysis

All experiments were performed in triplicates and data are presented as mean ± standard deviation (SD). Student’s *t* test and Chi-square test were used as appropriate in calculating the difference between groups. *P* value < 0.05 was considered to be statistically significant.

## Supplementary information

supplementary figure 1

supplementary figure 2

supplementary figure legends
