# Supplementary figures and images for: Long non-coding RNA NEAT1 confers oncogenic role in triple-negative breast cancer through modulating chemoresistance and cancer stemness
Source: Cell Death Dis. 2019 Mar 20;10(4):270. doi: 10.1038/s41419-019-1513-5 (PMC6426882; doi:10.1038/s41419-019-1513-5)

## Slide 1
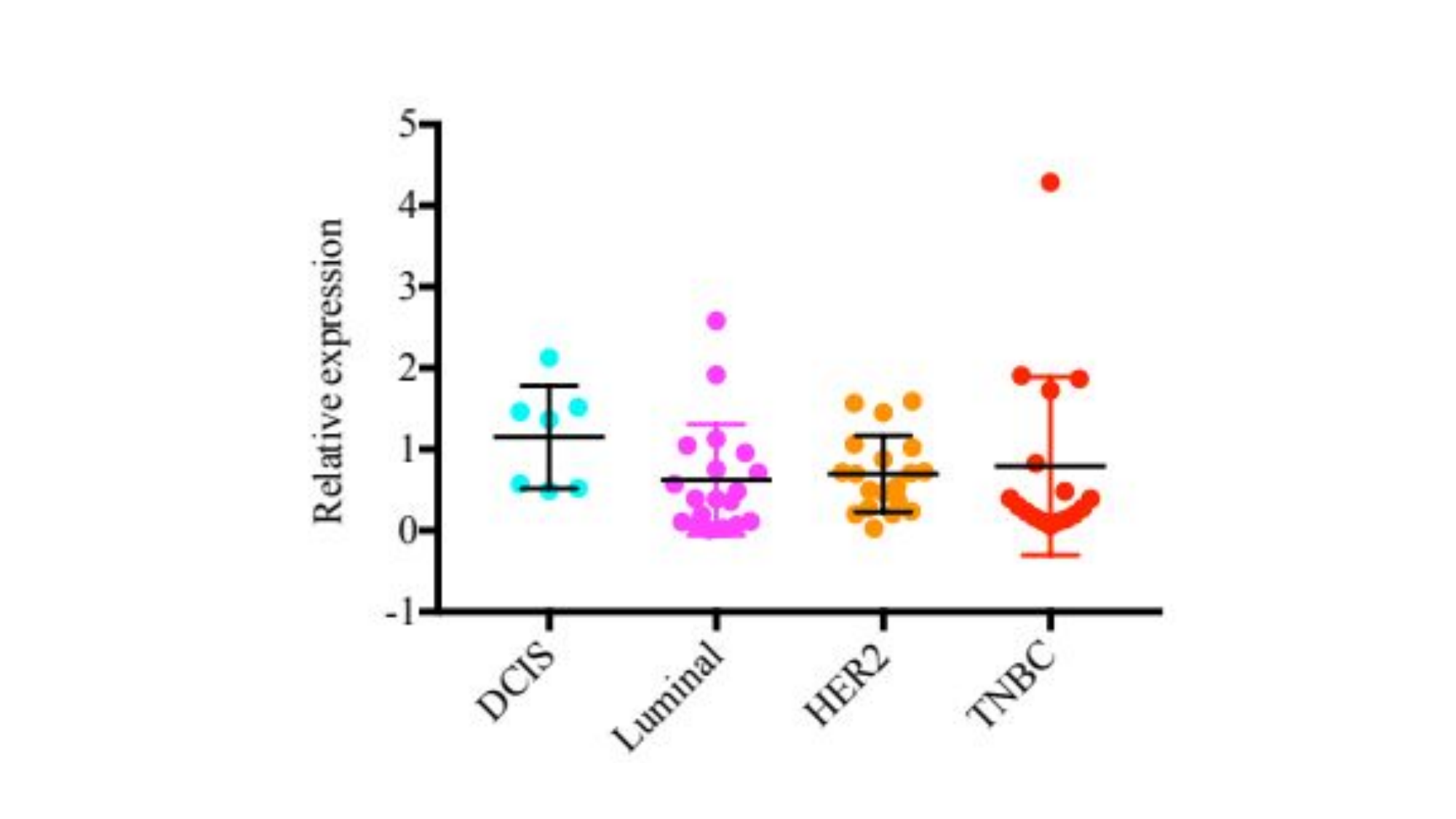

Supplement: Supplementary file 1 — supplementary figure 1 [file 41419_2019_1513_MOESM1_ESM.pptx]

## Slide 1
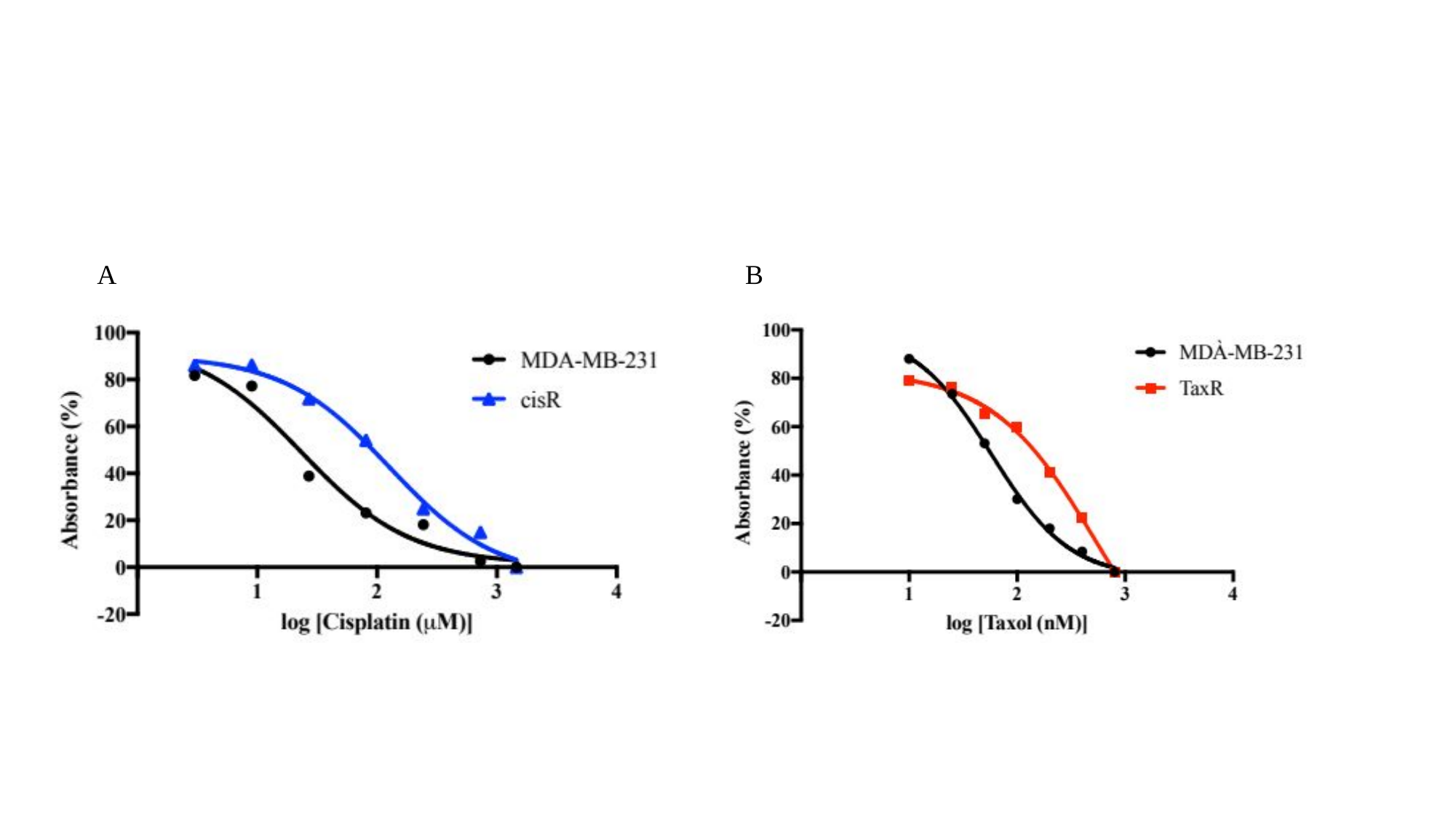

A
B

Supplement: Supplementary file 2 — supplementary figure 2 [file 41419_2019_1513_MOESM2_ESM.pptx]
