# Supplementary material for: Long non-coding RNA NEAT1 confers oncogenic role in triple-negative breast cancer through modulating chemoresistance and cancer stemness
Source: Cell Death Dis. 2019 Mar 20;10(4):270. doi: 10.1038/s41419-019-1513-5 (PMC6426882; doi:10.1038/s41419-019-1513-5)
Supplement: Supplementary file 3 — supplementary figure legends [file 41419_2019_1513_MOESM3_ESM.docx]

Supplementary Fig 1. NEAT1 isoform 2 expression among DCIS, Luminal, HER2 and TNBC subtypes.

Supplementary Fig 2. IC_50_ curves of cisplatin (A) and taxol (B) in MDA-MB-231, cisR and taxR cells.
